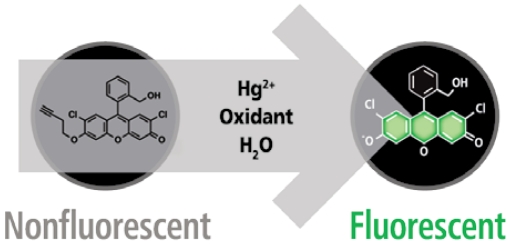# INNOVATIVE TECHNOLOGIES: Fluorescent Probe Detects Mercury

**Published:** 2009-02

**Authors:** Carol Potera

Mercury is toxic to the nervous system and may cause memory loss, cognitive and behavioral disturbances, headaches, insomnia, peripheral nerve damage, tremors, and motor dysfunction—all problems that have been observed to disappear upon removing the source of mercury exposure, according to the International Programme on Chemical Safety. Removing the source may now become easier with a new chemical probe that gives off a bright green glow when it reacts with mercury in fish and dental amalgams. Eventually the probe could be used to detect contaminated fish at home or mercury in wastewater at dental offices.

Developer Kazunori Koide, an associate professor in the University of Pittsburgh Department of Chemistry, based the probe on a simple principle taught in undergraduate organic chemistry classes: Mercury converts alkynes into ketones. Koide’s team synthesized an alkyne-based compound from commercial dichlorofluorescein. When mercury converts the alkyne into a ketone, the reaction generates a green fluorescent signal whose intensity corresponds to the amount of mercury present. “It’s a very straightforward approach,” Koide says, “yet no one ever thought to do this.”

The probe performed well in real-life tests described in the 10 December 2008 issue of the *Journal of the American Chemical Society*. A piece of salmon about the size of a dime was first treated with an oxidizing agent to release mercury bound up as methylmercury, then was soaked in the alkyne test solution. Within 30 minutes, mercury in the sample had converted the test solution to a ketone, which produced a strong fluorescent signal. Next, the researchers soaked a thin cloth with saliva and pressed it against an extracted tooth filled with silver amalgam (which may contain up to 50% mercury) obtained from a dental clinic. When the solution was applied to the cloth, its green fluorescent signal was substantially brighter than that of a control cloth also treated with saliva but not held against the tooth.

Because the sulfur-containing amino acid cysteine is known to bind tightly to mercury in fish, Koide wondered whether cysteine in sulfur-rich foods such as onions and eggs would react with mercury in dental amalgams. In another experiment, two extracted teeth containing amalgams were soaked for an hour in a cysteine solution. The filled teeth released a significant amount of mercury into the cysteine solution as compared with teeth that had been soaked in water at a temperature of 35ºC.

Other probes for mercury reported in the scientific literature are largely based on chemical reactions between mercury and sulfur groups. However, sulfur-based tests are prone to oxidation during storage, making them impractical for environmental testing. The standard method for detecting mercury in environmental samples—atomic absorption spectrometry—is expensive. “We’re not trying to compete with atomic absorption spectrometry,” says Koide. “We want to fill a gap by making a probe that the general public can use.”

Koide’s fluorescent probe “provides a clever chemical approach for detecting mercury in complex samples,” says Chris Chang, an assistant professor of chemistry at the University of California, Berkeley. “The simplicity and sensitivity of this assay are promising for further application.” Koide and colleagues hope to turn this chemical tool into a kit, and are modifying the method to make it consumer-friendly, such as finding safe chemicals to transform extremely toxic methylmercury in fish into less toxic mercury species before testing.

## Figures and Tables

**Figure:**